# Supplementary figures and images for: Clarification of fibrin generation and degradation reaction processes of clot-fibrinolysis waveform in hemorrhagic disorders
Source: PLoS One. 2025 Aug 18;20(8):e0320111. doi: 10.1371/journal.pone.0320111 (PMC12360562; doi:10.1371/journal.pone.0320111)

2022.3.18

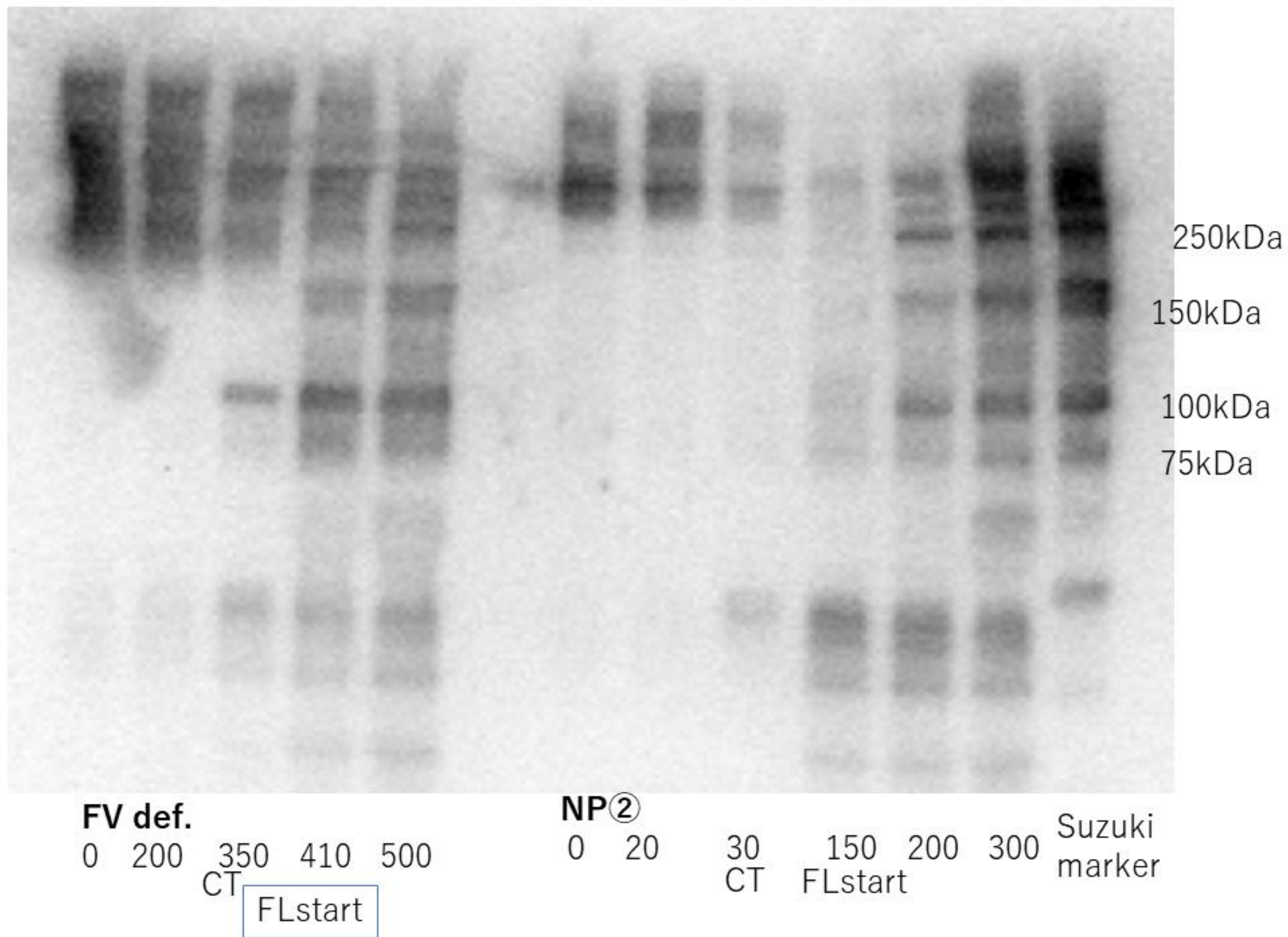

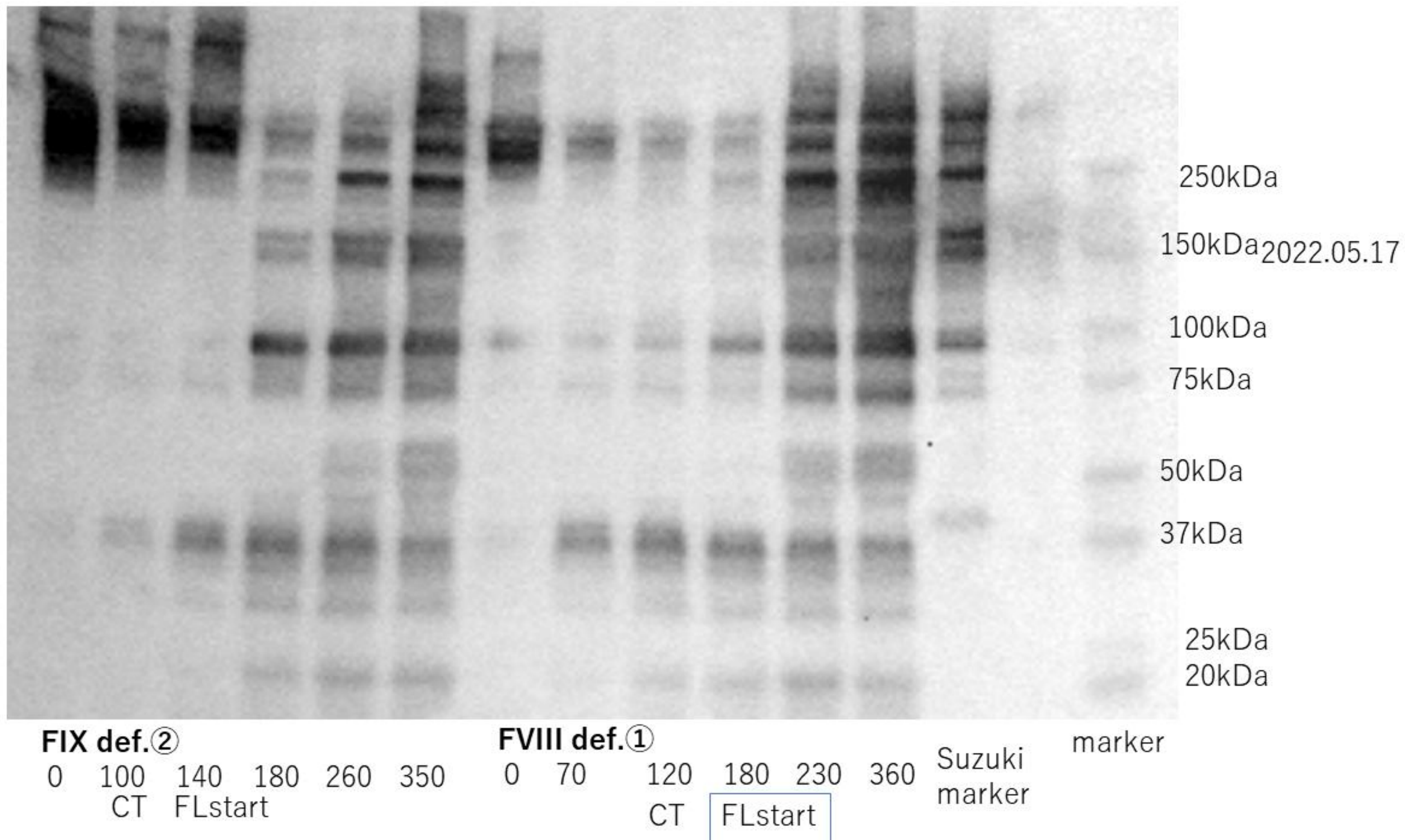

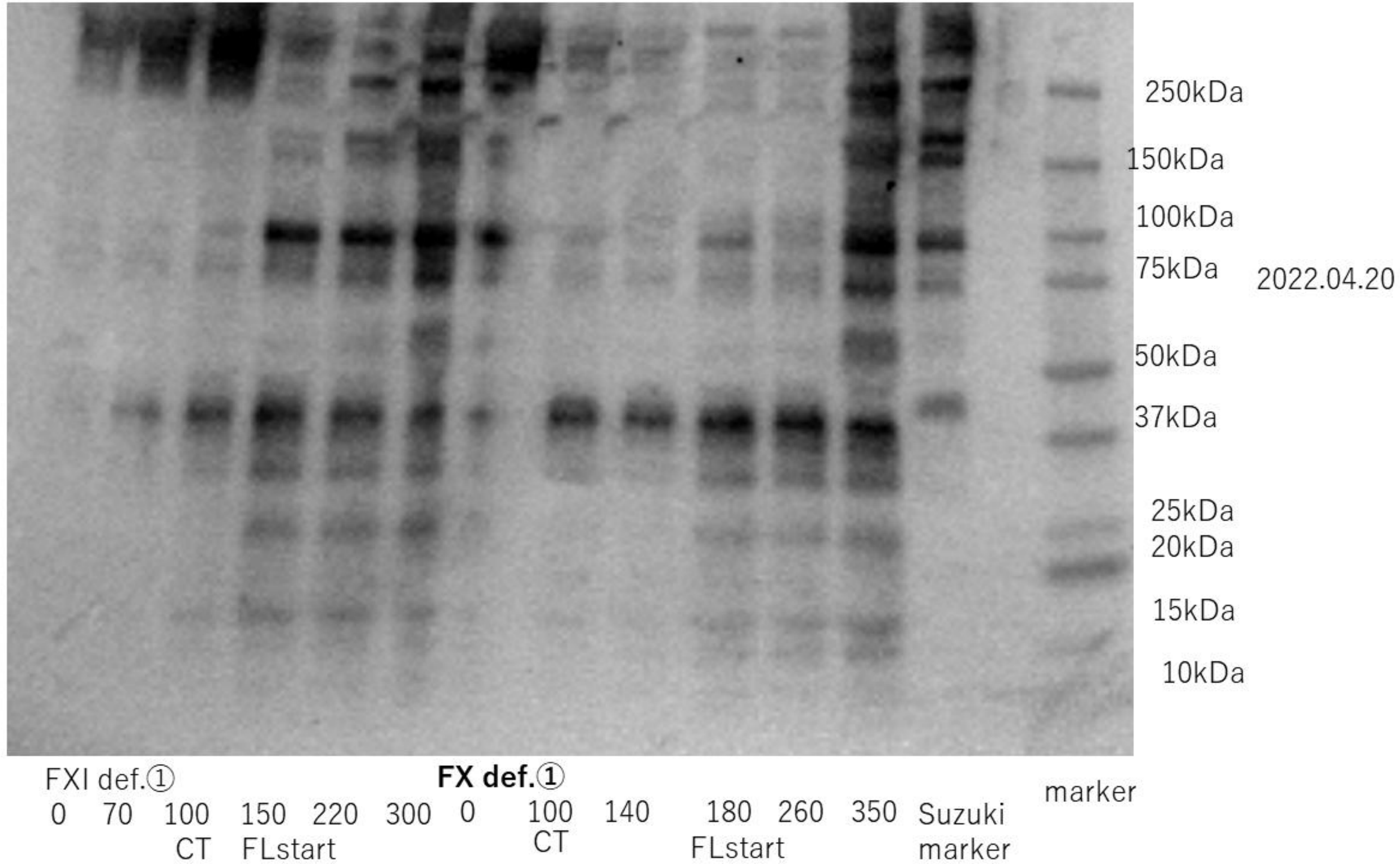

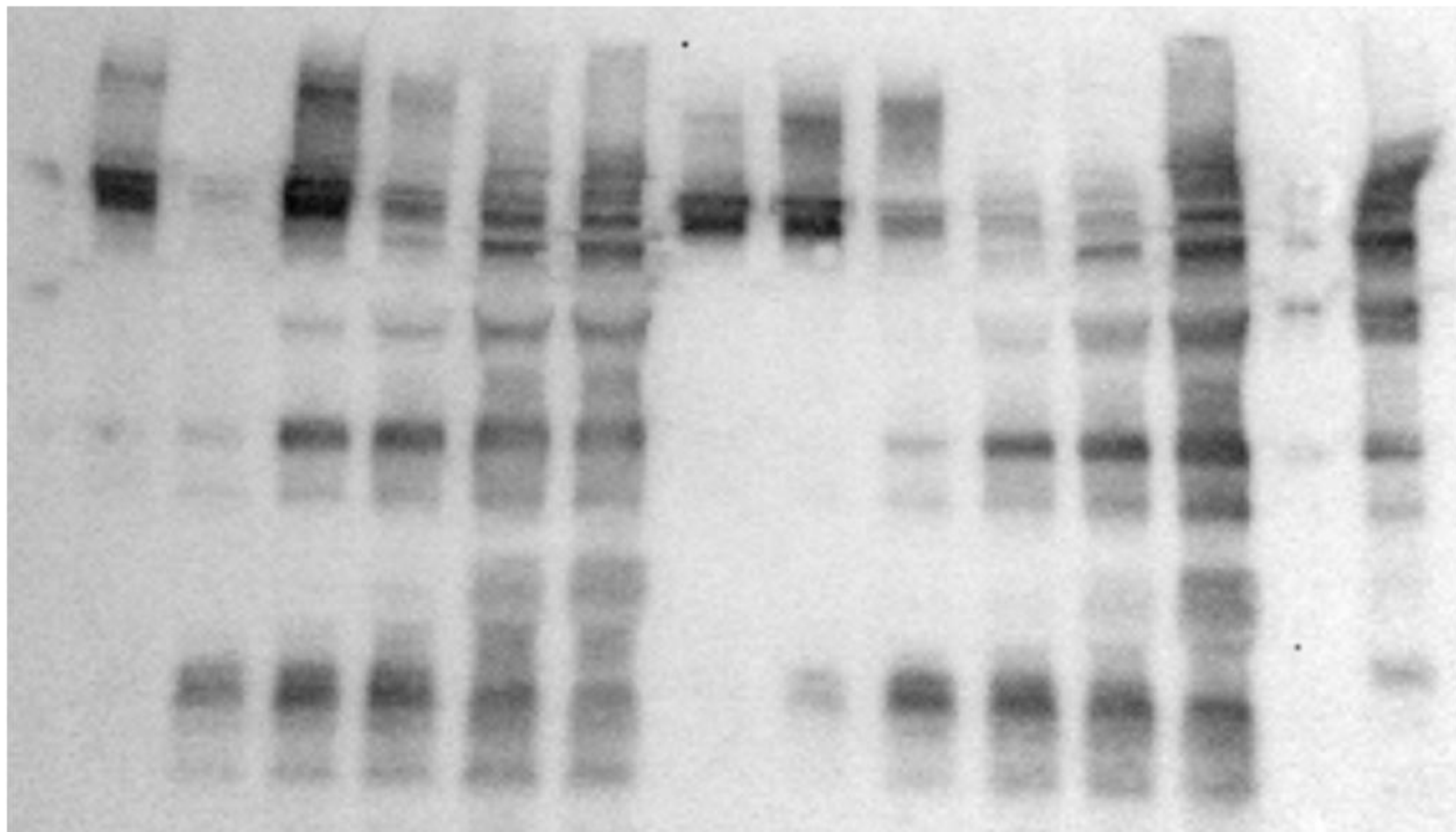

2022.05.17

FX def.②  
0 100  
CT

140 180 260 350  
FLstart

FXI def.②  
0 70 100  
CT

150 220 300  
FLstart

Suzuki  
marker

Supplement: S1 Fig — (PDF) [file pone.0320111.s001.pdf]

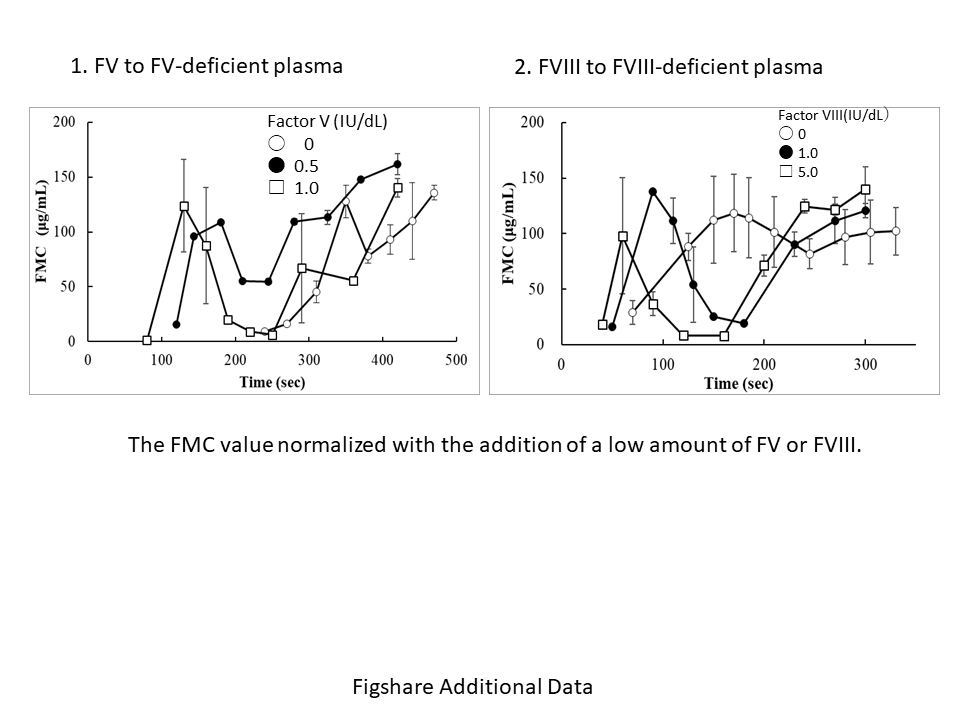

Supplement: S2 Fig — This figure presents FMC values in FV- and FVIII-deficient plasma with factor supplementation. (TIF) [file pone.0320111.s002.TIF]

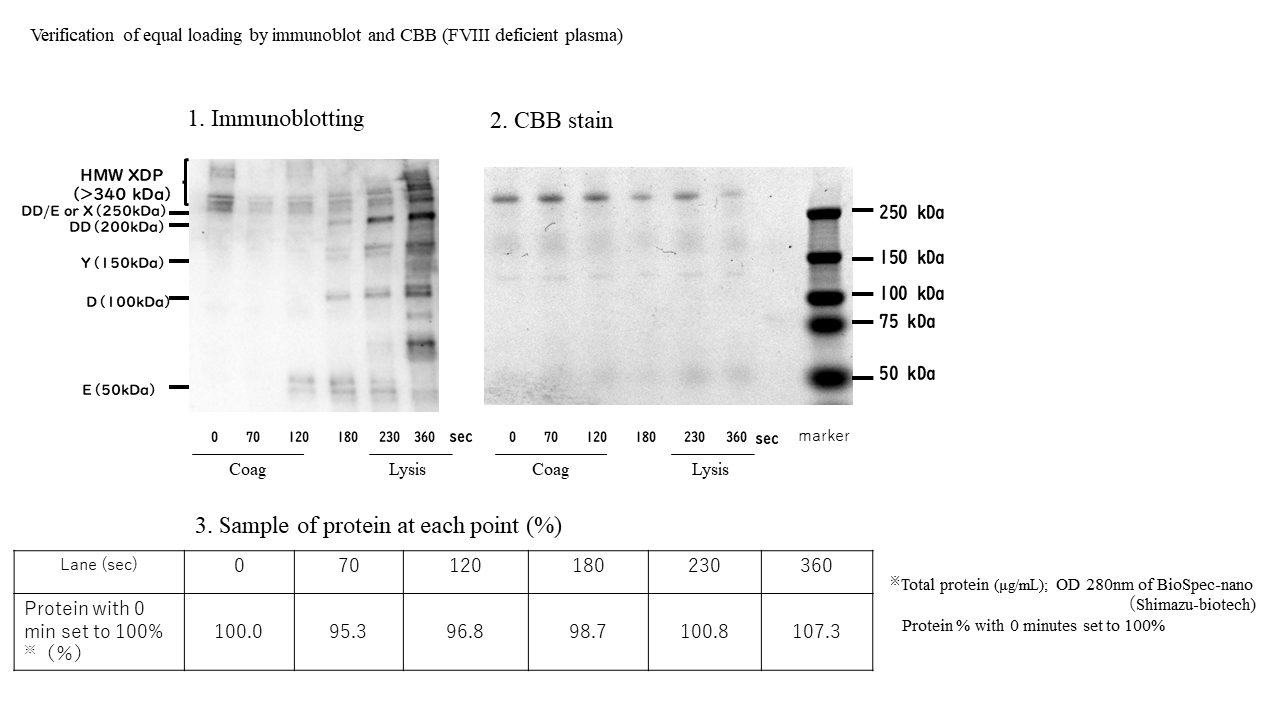

Supplement: S3 Fig — This figure includes supplementary results for fibrinogen and fibrin degradation analysis. (TIF) [file pone.0320111.s003.TIF]
