# Supplementary material for: Clarification of fibrin generation and degradation reaction processes of clot-fibrinolysis waveform in hemorrhagic disorders
Source: PLoS One. 2025 Aug 18;20(8):e0320111. doi: 10.1371/journal.pone.0320111 (PMC12360562; doi:10.1371/journal.pone.0320111)
Supplement: Attachment — Submitted filename: renamed_06fa8.docx [file pone.0320111.s005.docx]

Reviewer's Responses to Questions

Comments to the Author

Reviewer #1: Comments to the Authors

The aim of the present study is to determine whether transmittance changes in CFWA reflect coagulation and fibrinolysis reaction by comparing them with other methods. The authors also compared normal plasma with coagulation factor-deficient plasma samples in CFWA. This study provides a valuable contribution to evaluating CFWA as a clinical application using basic protein assays.

1. The authors used plasma samples provided by company. To detect statistical differences, all experiments were performed three times. However, the authors conducted three experiments using only one sample of each plasma, which suggests that the authors assessed the error within the experimental procedures. The authors should use different lot samples or

We would like to thank you for your comments.

This article used commercially available plasma. We believe that the experiments necessary for investigating the tendency of CFWA were conducted using a single lot. Plasma deficient in factor VIII was tested in three other lots. The reason we selected factor VIII-deficient plasma is because of the severity of bleeding symptoms and the high proportion of patients with hemophilia A in congenital bleeding disorders. The results were similar to those reported in the paper.

However, we agree with you that it would be a good idea to conduct further studies using plasma from other lots or patient plasma. We have added the following to the end of the discussion section.

P.12; lane.34 “We will have to investigate the results using patient plasma in the future.”

2. The authors used rabbit anti-fibrinogen antibody provided by MP Biomedicals for immunoblotting. However, I could not find such an antibody on the MP Biomedicals web site. If the antibody was prepared by the authors, the authors should cite a reference that previously used this antibody. If this antibody is being reported for first time, the authors should provide additional data demonstrating its specificity for fibrinogen, including which part of fibrinogen it detects and so on, as this is critical for this study.

We would like to thank you for your comments.

The reagent was not labeled.

P.6; lane 35 We added the label. This experiment used Polyclonal rabbit anti-human fibrinogen (A0080) from Cite Ab.

3. In the immunoblotting assay, some lanes appear to contain different sample amounts. The authors should include an internal control (e.g., transferrin) or, at the very least, stain the membrane with Coomassie or ponceau S to confirm equal loading.

We would like to thank you for your comments.

In the Immunoblot, we added the same amount of sample to all lanes. We measured the same marker as an internal control in each gel. Furthermore, when examining various deficient plasma, we always performed Immunoblot experiments using pool normal plasma as a control for each measurement.

P.6; lane 29 “We added equal volumes to the gel.”

4. In general, alteplase is used at concentration of approximately 10 -100 nM for in vitro clot lysis assays. Is the amount of r-tPA used in this experiment appropriate?

We would like to thank you for your comments.

As you pointed out, the tPA concentration in CFWA is higher than the therapeutic concentration. CFWA is a method developed by Nogami, K., Matsumoto, T., et al. et al.: A novel simultaneous clot-fibrinolysis waveform analysis for assessing fibrin formation and clot lysis in hemorrhagic disorders. Br J Haematol. 2019 Nov;187(4):518-529. doi: 10.1111/bjh.16111.　 This method reflects coagulation and fibrinolysis processes within 500 seconds, and can be measured quickly using an automatic analyzer.

5. In addition to alpha2 antiplasmin, PAI-1 also play a role in fibrinolysis and should be briefly mentioned in the discussion section.

We would like to thank you for your comments.

The following content has been added to the discussion.

P.11; lane 10 “The participation of both α2-antiplasmin and PAI-I performs an important role in the fibrinolytic system. In this study, we did not examine a PAI-I fibrinolytic factors in detail. In the future, it is essential to investigate the relationship with CFWA using assays that reflect α2-antiplasmin and PAI-I.”

6. P14, lane 6-7, N.D., note detected, probably not detected?

We have corrected the mistake.

7. P8, lane 22, FCM should be changed to FMC. The authors should confirm these detail again.

We have corrected the mistake.

Reviewer #2: In this manuscript, the authors compare the results of their already published assy, the clot-fibrinolysis waveform analysis (CFWA), that simultaneously evaluate coagulation and fibrinolysis in vitro, to the measurements of several specific proteins, relative markers of either coagulation or fibrinolysis.

The paper address some interesting questions but is purely descriptive. Also, the writing could be improved to make it easier to read.

I would have some major comments:

1/ For readers that are not initiated to CWA, it would be important to remind in the introduction that this global assay is performed on plasma and to compare it to whole blood global assays such as thromboelastography and to other plasma CLA (Pieters M, JTH 2019) or thrombin/plasmin generation fluorogenic assays. For example, why use kaolin as a trigger and not thrombin ?

Page 3. line 13: “The thromboelastography can evaluate coagulation and fibrinolysis, but it has low reproducibility (Nagler M,et.al, T＆H 2014). The clot lysis time (CLT) with thrombin addition can assess fibrinolysis, but it does not reflect intrinsic or extrinsic coagulation (Pieters M, et.al, JTH 2018). CFWA uses plasma as the measurement sample and an automated coagulation analyser, so results are available within 10 minutes and it has excellent reproducibility, and it can use plasma stored in a freezer. This investigation was performed using a modified APTT reagent to evaluate the bleeding tendency caused by a decrease in clotting factors, including haemophilia, which is an endogenous coagulation disorder.”

We have added the above text and references to the Introduction.

2/ Page 3, line 19: to assess DOAC in vitro effects is different from assessing their clinical efficacy. Please reformulate.

We would like to thank you for your comments.

Page3, line 26; efficacy→*in vitro* effects

We have made the changes you suggested.

3/ Figures 1A and B are redundant with 1C. Only 1C should be kept.

We would like to thank you for your comments.

We have combined Figure 1 into a single diagram.

4/ Figure legends should be separated from the main text and put at the end of the manuscript

We would like to thank you for your comments.

We have complied with the submission guidelines and prepared this paper, but we have moved the figure legend to the end of the main text as you suggested.

5/ Are PIC equivalent to PAP (plasmin-antiplasmin) complexes? If so, the name should be changed.

We would like to thank you for your comments.

As you pointed out, we changed the PIC to a PAP.

6/ It is unclear if all laboratory tests performed on CS-2400 analyzer included FDP, D-dimer and PIC. Also, the name of each commercial reagent kit should be added.

We would like to thank you for your comments.

We had already written this, but we have made it easier to understand. (Page 6, line 14-19)

7/ The intriguing result from this study is the increase of FMC during the fibrinolysis phase, indicating that FMC are generated during fibrinolysis or that the antibody used to measure FM also recognize small fragments released from FM fibrinolysis... It would have been interesting to compare FMC to thrombin-antithrombin complexes that would really reflect thrombin generation overtime.

Your point is entirely correct. We have added it. The comparison between FMC and TAT is a subject for the next time.

Page 9, line 24 “The increase in FMC in the fibrinolytic phase indicates that the antibodies used to measure FMC or to produce FMC recognize not only FMC but also small FMC fragments released from fibrinolysis.”

We have added the above text to the results.

8/ Also fibrinolysis markers increase earlier in deficient-plasma compared to normal plasma due to potential less quality fibrin fibers: is there any way to analyse fibrin density on CS-analyzers (from OD value delta at different wavelenghts?)

The automated coagulation analyser (CN-6000) reflected the fibrin density by measuring changes in turbidity of transmitted light. Reference 24 stated that when FVIII was added to factor VIII-deficient plasma (0-100 IU/dL), the transmitted light (％Transmittance) increased and the turbidity changes improved.　 This reference cites a report (Lilley L, et al. Res Pract Thromb Haemost. 2017;1:231-241) that found that the addition of FVIII increased the density of fibrin fibres, and that the fibrin clot fibres were composed of thinner, more highly branched fibres.　 We were able to analyse fibrin clots in the absence of clotting factors in detail in our study.

Page 11, line 36 “Lilley L et al. demonstrated that the addition of FVIII increased the density of fibrin fibres, and that the fibrin clot fibres were composed of thinner, more highly branched fibres (25) In addition, in the present study, by analysing the fibrinolytic reaction, it was considered that in plasma deficient in clotting factors, the density of fibrin fibres decreased, and the fibres were thicker and did not branch as much.”

The above text has been added to the discussion, and the references have also been added.

9/ Results from Western Blot and Figure 4 description should be moved to the Result section. Also the meaning of the different FDP (HMW XDP, DD, Y, D and E fragments) should be explained in the Figure legend.

We would like to thank you for your comments.

We have added an explanation of the molecular weight marker and FDP fragment fraction to Figure legend 4.

10/ The number of individual experiments or replicates should be specified at least in each figure/table legend.

We would like to thank you for your comments.

The number of times each item was examined has been added to the figure legend（n= ）.
