# Supplementary material for: Clarification of fibrin generation and degradation reaction processes of clot-fibrinolysis waveform in hemorrhagic disorders
Source: PLoS One. 2025 Aug 18;20(8):e0320111. doi: 10.1371/journal.pone.0320111 (PMC12360562; doi:10.1371/journal.pone.0320111)
Supplement: Attachment — Submitted filename: renamed_73ce7.docx [file pone.0320111.s006.docx]

Manuscript ID: PONE-D-25-07516R1

Title: Clarification of fibrin generation and degradation reaction processes of clot-fibrinolysis waveform in hemorrhagic disorders

We sincerely thank the Academic Editor and the reviewer for the thoughtful comments and suggestions regarding our revised manuscript. We have carefully addressed all remaining concerns and revised the manuscript accordingly. Below, we provide a point-by-point response to the comments.

Editor’s Comment:

The intriguing result from this study is the increase of FMC during the fibrinolysis phase, indicating that FMC are generated during fibrinolysis or that the antibody used to measure FM also recognize small fragments released from FM fibrinolysis... It would have been interesting to compare FMC to thrombin-antithrombin complexes that would really reflect thrombin generation over time.

This comment remains only partially addressed. Strengthening the discussion around this point, particularly regarding the interpretation of FMC levels and the potential value of TAT comparisons, would improve the clarity and impact of your findings.

Response:

We appreciate this important suggestion and have revised the discussion section to better clarify the interpretation of increased FMC levels during the fibrinolysis phase. Specifically, we now note the following:

Page 11, Lines 29-33；Thus, FMC may have the reactivity in fibrinolysis phase. It is also useful to measure thrombin-antithrombin complex (TAT) for more classification between coagulation and fibrinolysis phase reaction. Because TAT is considered to increase in only coagulation reaction. The relationship between TAT and FMC was reported in clinical study, which these markers showed similar tendency [24]. Another study to investigate the relationship is planned.

We believe these additions have improved the clarity and scientific context of our findings. Although it is difficult to compare FMC tendency with that of TAT due to the lack of several samples, we would like to conduct it as another separated study. Again, thank you for your suggestion.

Reviewer #1 Comments and Author Responses

Comment 1: In terms of question #2, the polyclonal anti-human fibrinogen antibody (A0080) is provided by Dako, not Cyte ab.

Response:

Thank you for pointing this out. We have corrected the manuscript to properly cite the antibody as:

Page 6, Lines 35–36； “Polyclonal rabbit anti-human fibrinogen ( Dako, Glostrup, Denmark)”

Comment 2: In some immunoblotting results, band intensities appeared to vary between lanes, despite equal loading of samples. To verify that the same amount of protein was loaded in each lane, immunoblotting for serum proteins such as albumin or transferrin should be performed. Alternatively, the transferred PVDF or nitrocellulose membrane should be stained with Ponceau S to assess total protein levels. Another option is to prepare duplicate gels, using one for immunoblotting and staining the other with Coomassie Brilliant Blue to confirm equal loading.

Response:

We have taken the reviewer’s advice and performed the suggested verification. Specifically, we used the third approach: preparing duplicate gels, with one used for immunoblotting and the other for Coomassie Brilliant Blue (CBB) staining. This was conducted using FVIII-deficient plasma samples.

Additionally, we measured the protein concentration at each point from 0 to 360 seconds using the BioSpec-nano (Shimazu-biotech) device with absorbance (280 nm) using the same sample as the immunoblot. The detailed methos were as follows.

First, O.D. 280 was detected in BioSpec-nano in each sample.

Second, the protein concentration was estimated from O.D. 280 in the analyzer. Coefficient of molar absorbance of human albumin was used for the calculation tentatively, and the tentative concentration was obtained.

Third, the concentration was compared among the samples. The concentration of Lane 1 (Time 0) was defined as 100%. Each protein concentration value was shown as relative value against sample of Lane 1. Relative value (%) = Concentration of each sample / concentration of Lane 1 (Time 0)

The range of protein concentration was 95.3-107.3% among 6 samples. The variability was within 10%, indicating that the protein concentration was the same level among the samples. Therefore, we suggested that the immunoblotting results reflected the change of fibrinogen/fibrin degradation.

Furthermore, the CBB-stained gel showed comparable band volume values from 0 to 120 seconds, indicating consistent protein loading. After 180 seconds, a decrease in band volume was observed, which we attribute to the degradation of fibrin degradation product (FDP) fractions. These results support the accuracy of our immunoblotting data and the observed time-dependent changes in protein levels. For the detailed data, please find the “Immunoblot and CBB 1,2” files.

We thank the reviewer and editor again for their guidance, which helped us strengthen our manuscript.

Sincerely,

Matsumoto Tomoko., Ph.D.,

Faculty of Health Care, Department of Clinical Laboratory Science, Tenri University,

80-1 Bessho-cho, Tenri, Nara 632-0018, Japan.

Tel.: +81-743-63-7811; Fax.: +81-743-63-6211;

E-mail: t.matsumoto@sta.tenri-u.ac.jp

On behalf of all co-authors
